# The Effectiveness of eHealth Technologies on Weight Management in Pregnant and Postpartum Women: Systematic Review and Meta-Analysis

**DOI:** 10.2196/jmir.8006

**Published:** 2017-10-13

**Authors:** Diana Sherifali, Kara A Nerenberg, Shanna Wilson, Kevin Semeniuk, Muhammad Usman Ali, Leanne M Redman, Kristi B Adamo

**Affiliations:** ^1^ School of Nursing Faculty of Health Sciences McMaster University Hamilton, ON Canada; ^2^ Clinical Nurse Specialist Hamilton Health Sciences Hamilton, ON Canada; ^3^ Department of Medicine University of Calgary Calgary, AB Canada; ^4^ School of Human Kinetics Faculty of Health Sciences University of Ottawa Ottawa, ON Canada; ^5^ Department of Clinical Epidemiology and Biostatistics Faculty of Health Sciences McMaster University Hamilton, ON Canada; ^6^ Reproductive Endocrinology and Women's Health Lab Pennington Biomedical Research Center Louisiana State University Baton Rouge, LA United States; ^7^ Department of Pediatrics Faculty of Medicine University of Ottawa Ottawa, ON Canada

**Keywords:** eHealth, technology, pregnancy, postpartum, weight

## Abstract

**Background:**

The emergence and utilization of electronic health (eHealth) technologies has increased in a variety of health interventions. Exploiting the *real-time* advantages offered by mobile technologies during and after pregnancy has the potential to empower women and encourage behaviors that may improve maternal and child health.

**Objective:**

The objective of this study was to assess the effectiveness of eHealth technologies for weight management during pregnancy and the postpartum period and to review the efficacy of eHealth technologies on health behaviors, specifically nutrition and physical activity.

**Methods:**

A systematic search was conducted of the following databases: MEDLINE, EMBASE, Cochrane database of systematic reviews (CDSR), Cochrane central register of controlled trials (CENTRAL), CINAHL (Cumulative Index to Nursing and Allied Health Literature), and PsycINFO. The search included studies published from 1990 to July 5, 2016. All relevant primary studies that involved randomized controlled trials (RCTs), non-RCTs, before-and-after studies, historically controlled studies, and pilot studies were included. The study population was adult women of childbearing age either during pregnancy or the postpartum period. eHealth weight management intervention studies targeting physical activity, nutrition, or both, over a minimum 3-month period were included. Titles and abstracts, as well as full-text screening were conducted. Study quality was assessed using Cochrane’s risk of bias tool. Data extraction was completed by a single reviewer, which was then verified by a second independent reviewer. Results were meta-analyzed to calculate pooled estimates of the effect, wherever possible.

**Results:**

Overall, 1787 and 176 citations were reviewed at the abstract and full-text screening stages, respectively. A total of 10 studies met the inclusion criteria ranging from high to low risk of bias. Pooled estimates from studies of the effect for postpartum women resulted in a significant reduction in weight (−2.55 kg, 95% CI −3.81 to −1.28) after 3 to 12 months and six studies found a nonsignificant reduction in weight gain for pregnant women (−1.62 kg, 95% CI −3.57 to 0.33) at approximately 40 weeks.

**Conclusions:**

This review found evidence for benefits of eHealth technologies on weight management in postpartum women only. Further research is still needed regarding the use of these technologies during and after pregnancy.

## Introduction

### Background

Mobile phones and other electronic health (eHealth) technologies are now ubiquitous in modern society, with over 90% of the Canadian population utilizing these continuously evolving technologies [1]. To put this in perspective, according to the United Nations (UN), of the world’s 7 billion people, 6 billion have mobile phones, whereas only 4.5 billion have access to toilets [2]. The recent emergence of mobile and other eHealth technologies has resulted in an increased use of these tools in health prevention–, promotion-, and cessation-based intervention frameworks for varied clinical areas such as smoking cessation or medication adherence [3,4], and in diverse populations [5-7]. The mobile phone–based approach to health care problems offers health care providers several advantages as it: (1) enables remote data transmission from a participant’s environment in an affordable and accessible manner, (2) reaches all segments of the population, including those of lower socioeconomic status, (3) can be semi- or fully-automated for efficient use of clinic resources, (4) can utilize a video or voice-over approach to communication for reducing barriers to access among those with lower literacy, and (5) can be delivered to people in any location with Wi-Fi service, making this approach viable even in rural areas. Finally, the availability, adaptability, and low cost of mobile technologies provide a promising format for delivering lifestyle intervention programs on a regular basis.

The global availability of mobile technologies [8] has created opportunities for mobile phones to potentially contribute to the United Nations Millennium Development Goals, advocated by the World Health Organization (WHO), of improving maternal and child health through the use of these emerging technologies in health care interventions [9]. More importantly, over 90% of millennial expectant women, between the ages of 18 and 32 years, in countries such as the United States, Canada, the United Kingdom, and China, were found to be regular mobile phone users [10,11], suggesting that these devices may offer an alternative approach for delivery of health-related information. Moreover, 96% of pregnant women in North America have indicated an interest in receiving guidance on prenatal care through their mobile phone [11], and 74% of postpartum women report interacting with weight management materials [12]. To date, however, there is a lack of comprehensive studies evaluating their impact during pregnancy or the postpartum period. Although not specifically focused on weight management, the Text4baby study, used a simple text messaging campaign aimed at changing attitudes and beliefs of economically disadvantaged pregnant women and new mothers [13]. The program was highly successful as measured by increased health literacy and preparedness for motherhood among participants. Widespread adoption of the Text4baby program following the initial evaluation suggests that such technologies have broad appeal and represent a viable model for delivery of interventions in the area of maternal and child health. To date, other interventions that have used mobile and other electronic technologies during these critical periods of a woman’s life have targeted clinical areas relating to breastfeeding and general health [14], but have not examined other health behaviors in this population.

Other important clinical areas that may benefit from eHealth interventions include weight gain during pregnancy and postpartum weight loss. Both gestational weight gain (GWG) and postpartum weight retention are key contributors to the intergenerational cycle of obesity and cardiometabolic risk in the mother [15,16]. Pregnant women who exceed recommended GWG targets place themselves and their offspring at an increased risk of serious perinatal and future health complications [17]. Not only are these women highly susceptible to gestational diabetes, preeclampsia and other antenatal complications, but they are also at an increased risk of postpartum weight retention [18,19], which ultimately leads to higher rates of postpartum maternal obesity in the long term. It is critical to note, however, that pregnant and postpartum women often report receiving limited, if any, information from their health care providers on weight management during pregnancy and postpartum periods [20-22]. In fact, many health care professionals feel ill-equipped to deliver such counseling [23]. Although considerable systematic review evidence indicates that lifestyle interventions can successfully manage GWG and postpartum weight retention [24-28], when delivered in a personalized fashion, such individualized interventions are generally expensive and may lack scalability from a public health perspective. Consequently, in-person, provider-based delivery of weight management interventions is impractical in current prenatal and postnatal care environments because of the associated strains on the health care system and lack of health care resources. As such, effective real-world solutions are urgently needed to address the needs of women who are seeking personalized support, information, and guidance to assist them with management of their weight, especially those who are receptive to novel technology-based approaches [29]. Whereas eHealth technologies offer the potential to serve as low-cost, widely-available therapeutic tools to support lifestyle interventions for weight management during the pregnancy and postpartum periods, there remains a paucity of data supporting their efficacy and effectiveness during these periods [30]. As such, before the development and widespread implementation of eHealth technologies, a rigorous evaluation of the effectiveness of this delivery modality for health care interventions is required.

### Objectives

The primary objective of this systematic review was to assess the effectiveness of eHealth technologies for managing weight (loss, gain, or maintenance) during pregnancy and the postpartum period. The secondary objectives were to assess the effectiveness of eHealth technologies on other clinical outcomes, including (1) glycemic parameters and (2) health behaviors (ie, nutrition and physical activity).

## Methods

This systematic review was conducted following the preferred reporting items for systematic reviews and meta-analysis (PRISMA) guidelines [31].

### Population

The population of interest included adult women of childbearing age (≥18 years) either during pregnancy or the postpartum period. Studies that did not explicitly specify the inclusion of pregnant or postpartum women were excluded.

### Interventions and Comparators

This review investigated eHealth weight management interventions with a specific goal of targeting either GWG during pregnancy or weight loss during the postpartum period. Eligible eHealth technologies included the following: mobile phone (text-messaging or short message service [SMS] or mobile phone app), Web-based, email, personal digital assistant, handheld computer, home computer, or tablet app. The intervention must have included a health behavior component (nutrition or physical activity) in the eHealth technology. A minimum intervention duration of 3 months was required. The environment where the eHealth technology was implemented (eg, home-based and prenatal clinic) was not an eligibility criterion. Three different reference groups were considered as comparators: (1) in-person interventions, (2) other health technology interventions, and (3) no intervention (ie, standard care or usual health care environment).

### Outcomes

The primary outcome was weight management with specific targets of GWG, measured in kilograms (kg) in pregnant women or weight loss (measured in kg) in postpartum women. In both populations, we also investigated changes in glycemic status (eg, fasting and 2-hour glucose levels), nutritional measures (eg, total energy intake), and physical activity (eg, minutes of physical activity).

### Study Design

All relevant primary studies that involved randomized controlled trials (RCTs), non-RCTs such as clinical controlled trials (CCTs), pre-post studies, historically controlled studies, and pilot studies were included. All other study designs were excluded. All study protocols without preliminary results for data extraction were also excluded.

### Databases and Search Criteria

A systematic computerized literature search was conducted of the following databases: MEDLINE, EMBASE, Cochrane database of systematic reviews (CDSR), Cochrane central register of controlled trials (CENTRAL), CINAHL (Cumulative Index to Nursing and Allied Health Literature), and PsycINFO. The search included studies published from 1990 to July 5, 2016. The starting year of 1990 was selected because of the rapid rise and acceptance of technological innovations after this date. No studies were excluded based on language. Reference lists and associated paper citations were reviewed to identify other potential eligible papers that may have been missed during the initial search. The search terms as designed for the MEDLINE database with medical subject headings (MeSH) and keyword searching are outlined in Multimedia Appendix 1. These terms were modified accordingly to search the other databases.

### Study Selection

After searching the selected databases using the predefined terms, all identified citations were retrieved and screened by 2 independent reviewers in two stages. In the first stage of titles and abstract eligibility, each citation was independently screened by at least one reviewer. If one assessor excluded the paper, the second reviewer analyzed and verified the validity of the exclusion. Any disagreements between reviewers were resolved with a third reviewer. In the second stage, the full-text papers of all included studies were retrieved and screened for eligibility by 2 independent reviewers. Similarly, any disagreement regarding the status of a full-text papers was resolved by a third reviewer. For all eligible full-text papers, data extraction was completed by a single reviewer using standardized data collection forms, which were then verified by a second independent reviewer.

### Data Extraction

Data extracted from the research included study, participant and intervention characteristics, and outcomes. Study characteristics were author, year, objective, design, setting, geographical region, period (ie, pregnancy or postpartum), duration of the intervention, sample size, participant inclusion or exclusion criteria, recruitment time points, methods of recruitment, details of the eHealth intervention and comparison, and statistical analyses used. Participant characteristics were age, pregnancy history, ethnicity, education, household income, and baseline anthropometric measurements. Intervention characteristics included type of eHealth technology, focus of the intervention (ie, nutrition, physical activity, or both), use of the eHealth intervention (ie, expected vs actual use), other components in addition to eHealth, communication strategy, detailed features, participant satisfaction, and participant- and investigator-reported benefits and limitations. Outcomes encompassed the type of assessment of outcomes (objective, subjective, or self-reported), clinical and laboratory measurements (baseline and end of study), and treatment effects.

### Assessment of Risk of Bias

For included studies, the Cochrane Collaboration’s risk of bias tool was used [32] to assess the level of potential bias for each study based on six main methodological domains, which included the following: sequence generation, allocation concealment, blinding, incomplete outcome data, selective outcome reporting, and other sources of bias. Using this tool, each domain was scored as a low, unclear, or high risk of bias. The overall risk level was categorized based upon all six domains.

### Data Analyses

To perform meta-analysis, immediate posttreatment data (means and standard deviations) were utilized for continuous outcome measures, whereas number of events or prevalence was utilized for binary outcomes. The DerSimonian and Laird random effects models with inverse variance (IV) weighting method were utilized to generate the summary measures of effect in the form of mean difference for the continuous outcome measures and odds ratio (OR) for dichotomous outcomes. Mean differences in change scores were calculated using change from baseline data (ie, mean difference between pretreatment (baseline) and posttreatment (final or end point) values, along with the standard deviation (SD) for both intervention and comparison groups). For secondary outcomes such as glycemic parameters, daily energy intake, and daily servings, forest plots were created but no pooled estimates were provided, as the units of measurement, direction of effect, and outcome measures differed across studies. The Cochran’s Q (alpha=.05) was employed to assess statistical heterogeneity, and I^2^ statistic was used to quantify the magnitude of statistical heterogeneity between studies where I^2^ >50% represented moderate and I^2^ >75% represented substantial heterogeneity across studies.

## Results

### Study Selection

**Figure 1:**
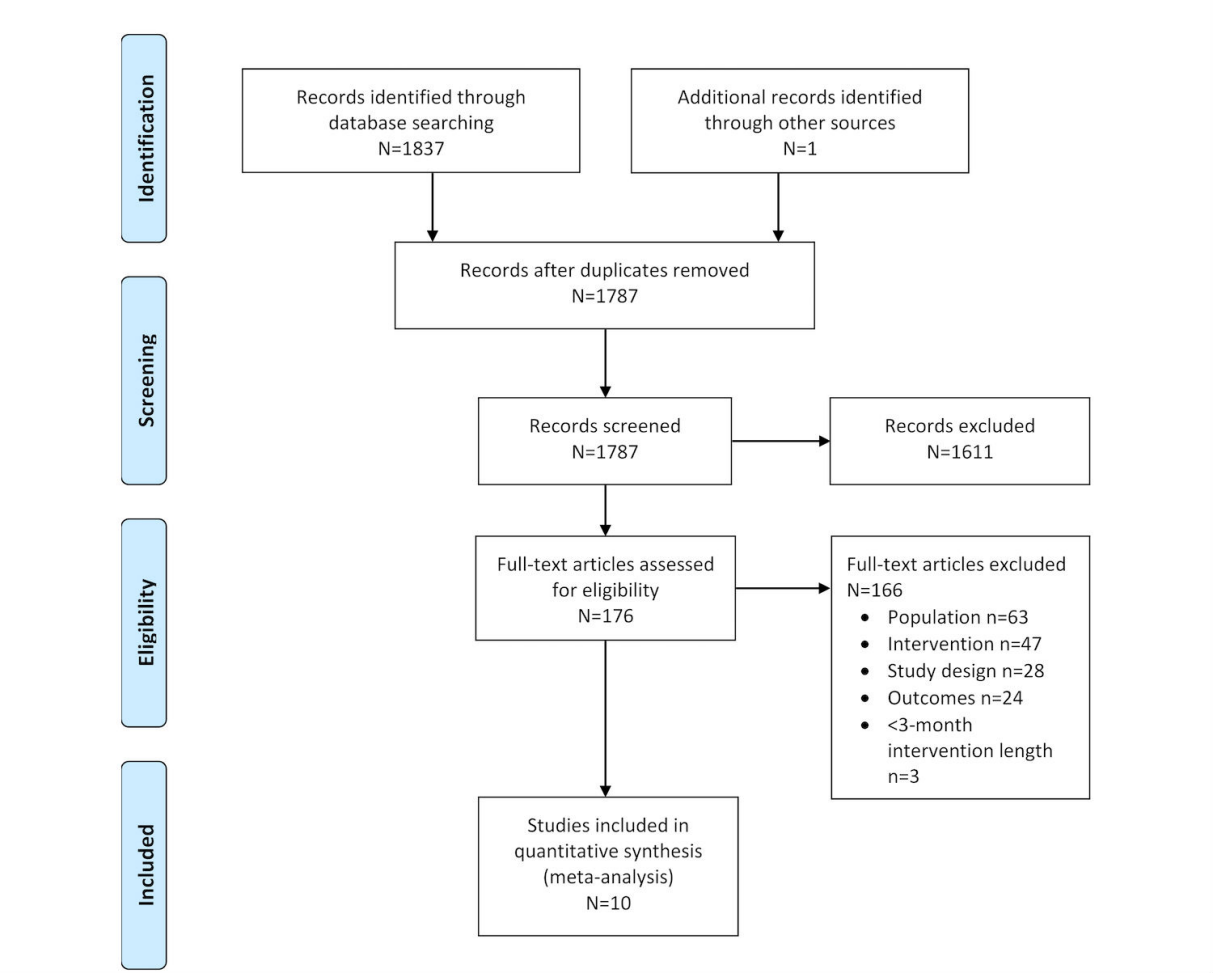
The preferred reporting items for systematic review and meta-analysis (PRISMA) flow diagram on the effectiveness of electronic health (eHealth) technologies for weight management in pregnant and postpartum women.

The literature search yielded 1837 citations from all of the databases, with one additional reference from gray literature (Figure 1; [31]). The removal of duplicate entries provided a total of 1787 citations. Next, 1611 citations were excluded after the screening of titles and abstracts, and an additional 166 citations were excluded at the full-text screening phase. In total, 10 studies (seven RCTs, one pilot RCT, and two CCTs) were included in the review.

### Study Characteristics

#### Participant Characteristics

Of the included studies, seven were conducted in the United States of America [33-39], two were from Spain [40,41], and one study was from the United Kingdom [42]. The number of participants within each of the studies ranged from 18 to 104, with a total sample size of 525 participants. The dropout/loss to follow-up rate ranged from 2.0% to 39.1% in the intervention groups and 0% to 25.0% in the control groups. The intervention group participants were aged between 24 and 36 years, whereas the participants from the control groups were aged between 24 and 35 years. Several of the included studies [33,35,36,39,40,41] provided measures of prepregnancy body mass index (BMI) with values ranging from 26 to 30 kg/m^2^ for the intervention groups and 25 to 30 kg/m^2^ for the control groups. Participant ethnicity varied between the studies and study arms (white: intervention: 12.5%-100%; control: 13.3%-100%) [33-36,38-42]. Several studies reported on the level of education within their population ranging from 78% of the total sample having a secondary degree [36], to other studies reporting approximately 20% or above in the usual care and 21% or above in the intervention having a postsecondary education [34,37,38,40,41]. Additional details on the characteristics of each of the included studies can be found in Multimedia Appendix 2.

#### Intervention Components

Six studies conducted the intervention during pregnancy [35-37,40-42], whereas four studies focused on the postpartum period [33,34,38,39]. Of the studies that provided interventions during pregnancy, several used common eHealth technological elements such as text messaging or website support. In particular, Pollak et al [35] used a text-based intervention targeting four health behavior goals during pregnancy, including: (1) targeted daily walking to 10,000 steps, (2) avoid sweetened drinks, (3) eat at least 5 fruits and vegetables each day, and (4) eliminate fast food intake. Only the first two goals were implemented during the initial stages of the intervention (approximately 10 weeks) with all four goals utilized for the rest of trial (approximately 6 weeks). Participants received targeted text messages each week with regard to their current goals and monthly text-message reminders on the Institute of Medicine’s (IOM) GWG guidelines. Carral et al [41] used a website specifically designed for monitoring people with diabetes during pregnancy that allowed for remote and bidirectional communication between health care professionals and patients, including relaying of messages and alerts for glucose monitoring. Herring et al [37] used text messaging, along with social media support groups and coaching to support women through nutritional and physical activity goals. The text messages were daily in frequency and personalized to each goal, building on skills and self-efficacy. The social media group was a forum to support and add further behavioral skills training. Perez-Ferre et al [40] used a telemedicine website and mobile phone app to support the transmission of glucose levels and for sending text messages. The website was used to monitor, adjust, and recommend insulin doses and goals. Smith et al [36] used a website that intervention arm participants would log on to review exercise and nutrition information. Specifically, this included recommendations, goal setting, problem-solving modules, a journal, a calendar, and a community forum for women to interact with other intervention arm participants. Finally, Soltani et al [42] used text messaging and self-monitoring diaries to support women through behavior modification for weight management, physical activity, and nutrition.

Of the studies that provided postpartum interventions, several eHealth strategies were used, including websites, biosensors/activity monitors (ie, pedometers), and text messaging. Colleran et al [33] utilized a Web-based dietary intervention to reduce dietary intake by 500 kcal/day below calculated energy requirements and compared results with recommendations provided on a weekly basis, along with providing strategies to assist women in meeting their outlined recommendations. Kim et al [34] employed a structured Web-based physical activity intervention in which participants received a pedometer and access to a Web-based curriculum. Participants were also provided with personalized step count goals, strategies for meeting these goals, as well as the opportunity to anonymously interact with other intervention group participants through a Web-based study-specific forum. Nicklas et al [38] modified the diabetes prevention program (DPP) to 12 core modules that provided women with the opportunity to track goals (ie, walking and weight), to share secure messages with health care professionals, and to view Web-based media files. Finally, Herring et al [39] piloted a Web-based and text messaging intervention that focused on six empirically tested weight-related behavior change strategies and monitored women via text messaging.

Among studies, the intervention content was provided at varying frequencies, including: daily [34,37], 3 times per week [35], weekly [33,38,40,42], every 2 weeks [39,41], or on an individualized basis [36]. All of the eHealth technologies employed a bidirectional communication modality with asynchronous or interactive communication between the health care professionals and participants. The duration of the interventions in the pregnancy interventions ranged from 6 to 26 weeks, whereas the postpartum interventions ranged from 23 to 52 weeks. All comparator or control groups received usual standard of care or a simplified educational version of the technology offered to the intervention group, which provided only general health information.

### Risk of Bias in Included Studies

The results of risk of bias were determined using Cochrane Collaboration’s risk of bias tool for the 6 methodological domains and the overall risk level (Table 1). Of the included studies, the overall risk of bias for seven studies was rated with an unclear risk of bias [33-35,36,38-40], two studies were rated with a high risk of bias [41,42], and one study was deemed to have low risk of bias [37].

**Table 1:** Risk of bias for included studies.

Study (year), country	Risk of bias					
	Sequence generation	Allocation concealment	Blinding of participants/ personnel	Selective reporting	Other	Overall
Carral (2015), Spain [41]	High	High	High	Low	Low	High
Colleran (2012), United States of America [33]	Unclear	Unclear	Unclear	Low	Unclear	Unclear
Herring (2014), United States of America [39]	Low	Low	High	Low	Low	Unclear
Herring (2016), United States of America [37]	Low	Low	Unclear	Low	Low	Low
Kim (2012), United States of America [34]	Unclear	Low	Low	Low	Low	Unclear
Nicklas (2014), United States of America [38]	Unclear	Low	Low	Low	Unclear	Unclear
Pérez-Ferre (2010), Spain [40]	Unclear	Unclear	Unclear	Low	Low	Unclear
Pollak (2014), United States of America [35]	Unclear	Unclear	Unclear	Low	Low	Unclear
Smith (2016), United States of America [36]	Low	Unclear	High	Low	Low	Unclear
Soltani (2015), United Kingdom [42]	High	High	High	Low	High	High

### Synthesis of Results

#### Primary Outcome (Weight Management and Body Mass Index)

**Figure 2:**
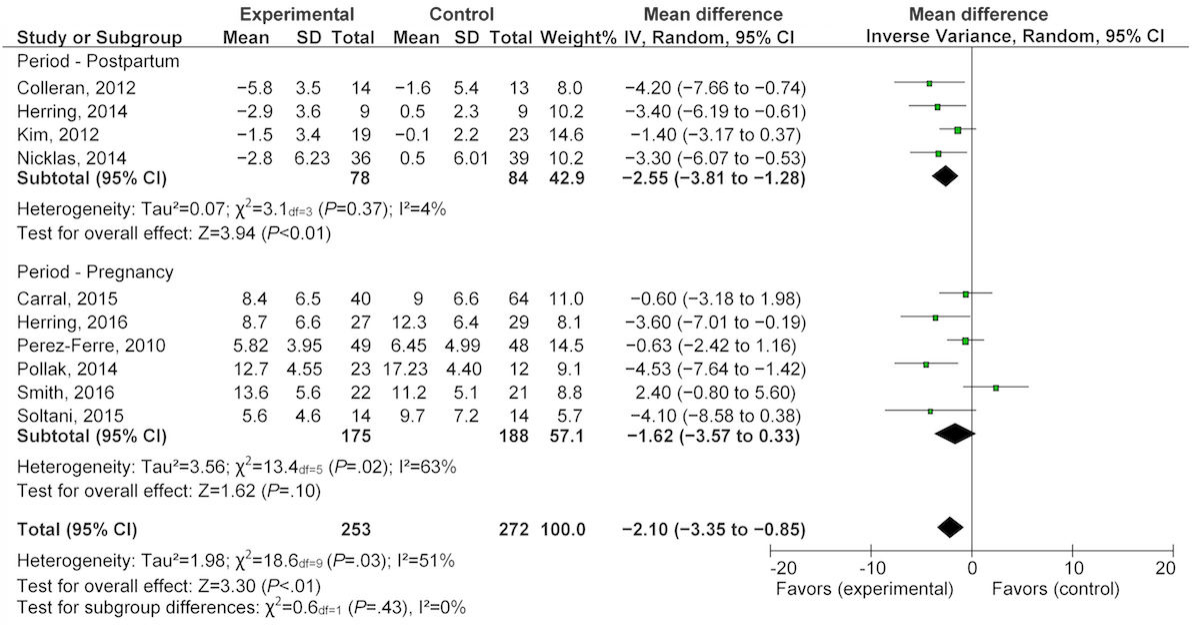
Pooled analysis of eHealth technologies on weight management (kg) in pregnant and postpartum women.

**Figure 3:**
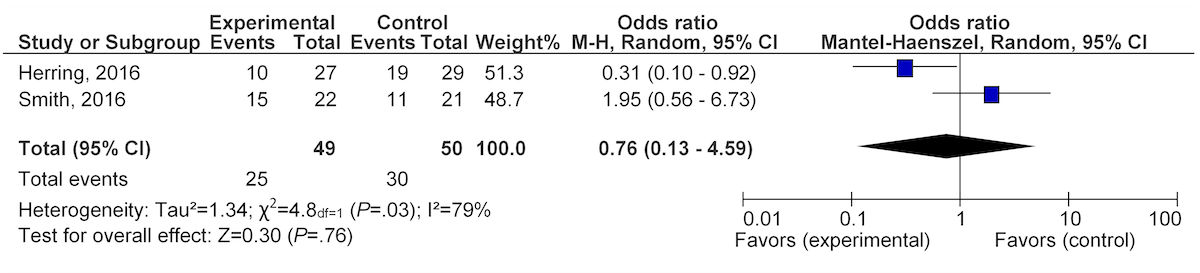
Pooled analysis of eHealth technologies on percentage of women gaining weight above IOM recommendations for pregnancy.

**Figure 4:**
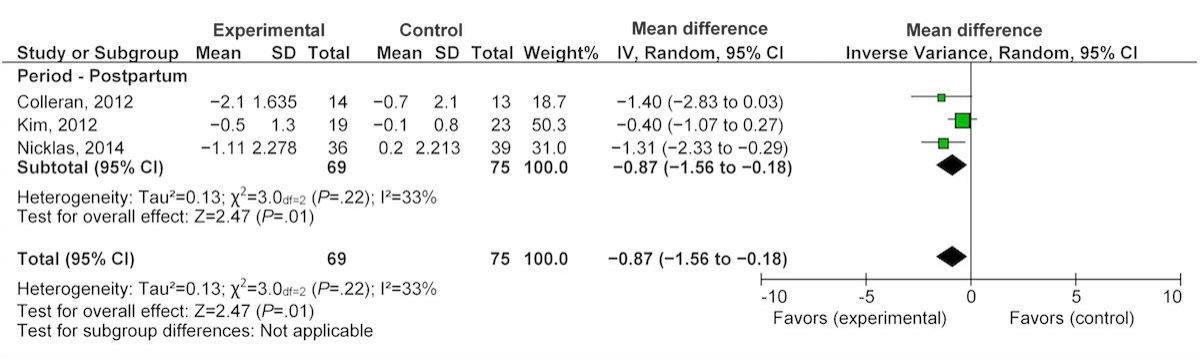
Pooled analysis of eHealth technologies on body mass index (kg/m^2^) in postpartum women.

All 10 studies reported on participant weight management in terms of weight gain, loss, or maintenance. During pregnancy, six studies [35-37,40-42] that evaluated eHealth technology for weight management found a nonsignificant reduction in GWG, with a mean difference of −1.62 kg (95% CI −3.57 to 0.33) after exposure to the intervention (Figure 2). Four studies contributed to the pooled analysis for the postpartum eHealth technology weight intervention, showing a statistically significant difference in weight loss, with a mean difference of −2.55 kg (95% CI −3.81 to −1.28) after completing eHealth weight management interventions (Figure 2) [33,34,38,39]. The overall pooled analysis for any eHealth technology intervention in the combined population of interest resulted in a statistically significant reduction in weight, with a mean difference of −2.1 kg (95% CI −3.35 to −0.85; Figure 2). When examining the percentage of women gaining weight above recommendations, two studies [36,37] provided a nonsignificant OR of 0.76 (95% CI 0.13 to −4.59; Figure 3). However, the change in BMI in the pooled postpartum studies [33,34,38] showed a significant reduction with a mean difference of −0.87 kg/m^2^ (95% CI −1.56 to 0.18; Figure 4).

#### Secondary Outcomes (Glycemic, Nutrition, and Physical Activity Parameters)

**Figure 5:**
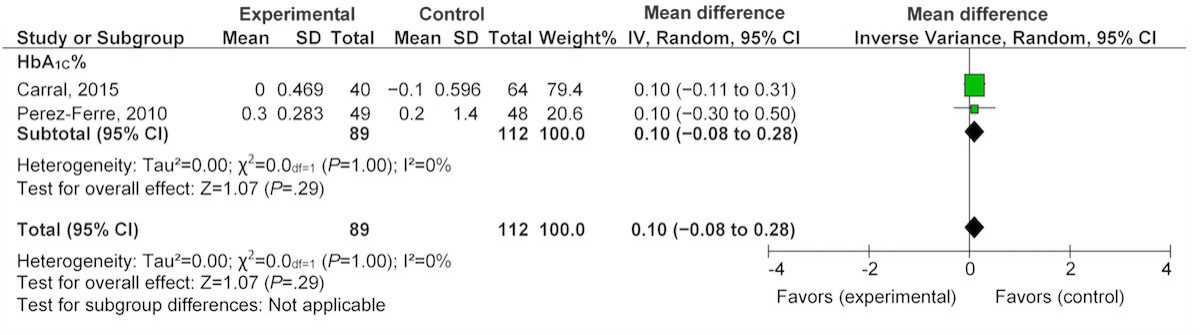
Effect of eHealth technologies on glycemic parameters in women during pregnancy.

**Figure 6:**
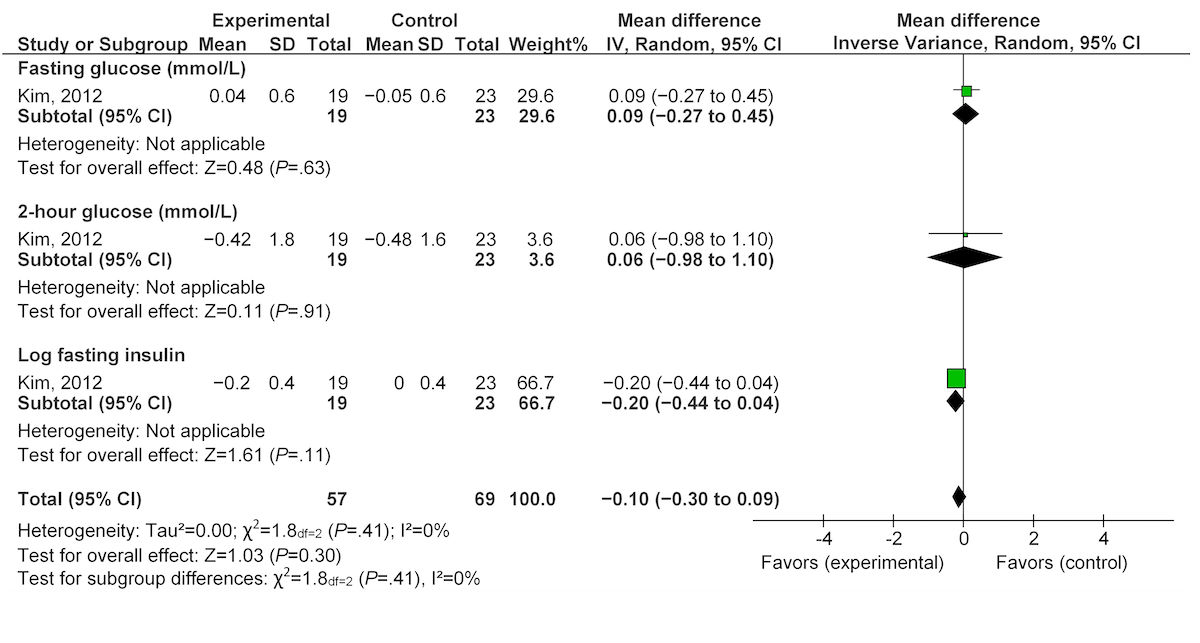
Effect of eHealth technologies on glycemic parameters in postpartum women.

Three studies provided data for changes in glycemic parameters, two studies during pregnancy [40,41], and one study postpartum [34]. The pooled change in glycemic parameters during pregnancy as measured by glycated hemoglobin (HbA_1C_) was an increase of 0.10 (95% CI −0.08 to 0.28; Figure 5). One study [34] that reported on glycemic parameters (fasting, 2 hour glucose, log fasting insulin) found that technology raised fasting glucose nonsignificantly by 0.09 mmol/L (95% CI −0.27 to 0.45) and 2-hour postprandial glucose by 0.06 mmol/L (95% CI −0.98 to 1.10). Finally, log fasting insulin decreased by −0.20 (95% CI −0.44 to 0.04; Figure 6). All glycemic changes were not statistically significant.

**Figure 7:**
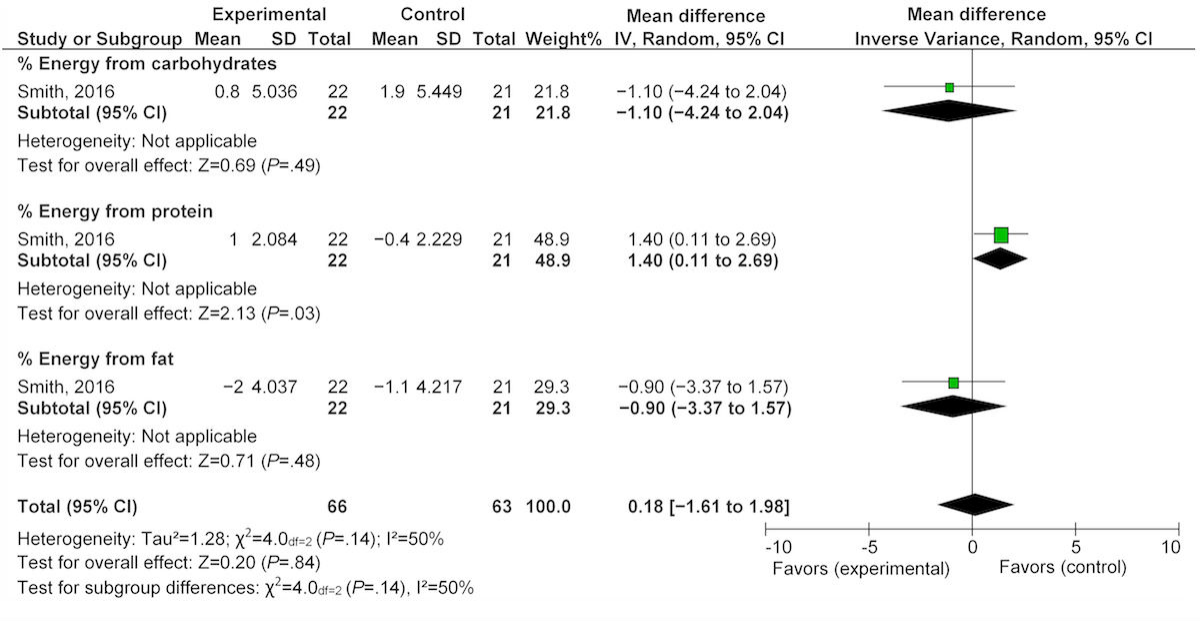
Effect of eHealth technologies on percentages of energy intake in women during pregnancy.

In addition, one study reported on nutrition status during pregnancy [36]. The study found that after exposure to a Web-based program, women reported a nonsignificant reduction in energy intake from carbohydrates (1.10%, 95% CI −4.24 to 2.04) and from fat (−0.90%, 95% CI −3.37 to 1.57), as well as a nonsignificant increase in energy intake from protein (1.40%, 95% CI 0.11-2.69; Figure 7).

**Figure 8:**
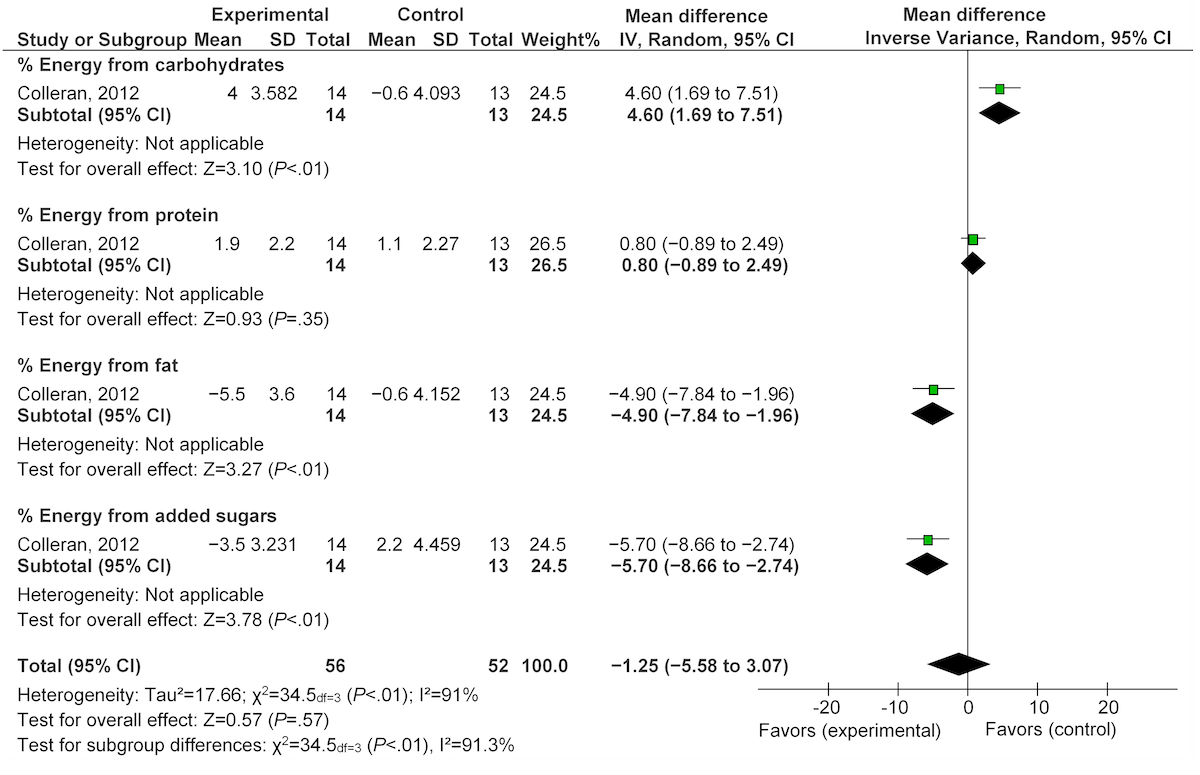
Effect of eHealth technologies on percentages of energy intake in postpartum women.

Another study reported on nutrition status changes postpartum after exposure to an eHealth technology intervention for 4 months [33]. This study found a statistically significant reduction in total daily energy intake of 442.0 kcal (95% CI −803.10 to −80.90). The same study found statistically significant reductions (Figure 8) in percentage of total daily intake of fat and added sugars by −4.90% (95% CI −7.84 to −1.96) and −5.70% (95% CI −8.66 to −2.74), respectively. Changes in the percentage of energy intake from carbohydrate significantly increased by 4.60% (95% CI 1.69-7.51), and the percentage of energy intake from protein decreased by −0.80% (95% CI −0.89 to 2.49), although this small change was not statistically significant (Figure 8). When examining daily servings of food groups, statistically significant reductions in the number of daily milk servings (−1.20, 95% CI −1.80 to −0.56) and daily servings of whole grains (−1.20, 95% CI −2.31 to −0.09) were noted [33]. However, daily servings of fruit, vegetables, oils or fat, and sweets were not significantly impacted by the 4-month exposure to the eHealth technology.

Finally, the pregnancy-specific study utilized the self-reported pregnancy physical activity questionnaire (PPAQ) for estimating physical activity levels during pregnancy [35]. No significant differences were found between the two groups in light or moderate physical activity as determined by the PPAQ at 32 weeks of gestation (moderate: 95% CI −3.5 to −0.3, *P*=.71; light: 95% CI −2.6 to 0.4, *P*=.08). A postpartum study that employed a Web-based self-report survey on physical activity habits [34] found no significant differences at baseline and follow-up with regard to the proportion of individuals within three activity categories (0 min/week, <60 min/week, and ≥60 min/week) for physical activity levels between the control and intervention groups, including any activity (baseline: *P*=.61; follow-up: *P*=.25), mild (baseline: *P*=.26; follow-up: *P*=.20), moderate (baseline: *P*=.81; follow-up: *P*=.51), and vigorous physical activity (baseline: *P*=.81; follow-up: *P*=.65). As a result of the heterogeneity of the measurement protocols between the pregnancy and postpartum studies, a meta-analysis was not conducted for the physical activity parameters.

## Discussion

### Principal Findings

#### Overall Effectiveness of eHealth Interventions in Pregnancy and the Postpartum Period

This review summarizes the most relevant/applicable trial evidence available to assess the effectiveness of eHealth technologies on weight management in pregnant or postpartum women. Notably, all of the included studies were recent (published between 2010 and 2016), which highlights the emerging use of this technology as a novel health care strategy, yet the low number of studies emphasizes the need for further evidence to support its efficacy for weight management in the clinical environment. To that end, the analysis of evidence within this review demonstrated that exposure to eHealth technology was associated with a nonsignificant benefit for weight management during pregnancy and a statistically and clinically significant weight reduction in the postpartum period. The pooled estimate for change in weight (kg) during pregnancy was −1.62 kg (95% CI −3.57 to 0.33), whereas the pooled estimate for change in weight postpartum was −2.55 kg (95% CI −3.81 to −1.28).

#### Components of Effective eHealth Interventions

The eHealth interventions that were effective in minimizing excessive weight gain (kg) during pregnancy comprised multiple components, including individualized text messaging and the use of social media [35,37]. Specific components that were effective in one study focused on a multimodal approach to eHealth, utilizing individualized text messaging for skills training and self-monitoring, private social media chat group for support, and individualized health coaching telephone calls [37]. Another study that was effective in reducing weight in pregnancy employed frequent text messaging (3 times per week), which focused on nutrition and physical activity by providing concise suggestions for modifying nutritional behavior (ie, avoid sweetened drinks) and increasing physical activity (ie, goal of 10,000 steps/day) [35].

Three studies demonstrated effectiveness in reducing postpartum weight with eHealth interventions. One study that included an eHealth solution (Web-based MyPyramid Menu Planner) with additional in-person counseling/support [33] had a greater reduction in weight and BMI as compared with the intervention that included only eHealth components (Web-based information, online forum, text messaging, and email) [34] implemented during the same period. Furthermore, another study found significant weight reduction when the eHealth intervention focused on both nutrition and physical activity (Healthy4Baby) [39]. Finally, one study modified the lifestyle-intensive DPP for postpartum women and also achieved a significant reduction in weight and BMI [38]. Overall, the multifaceted interventions (ie, targeting both physical activity and nutrition) with multiple and different modalities may be more effective than an eHealth-focused intervention targeting physical activity alone. More importantly, none of the studies performed an evaluation to separate the effects of personal contact with a health professional from the effects of the eHealth intervention alone. This information could help determine predictors of participant engagement or adherence with the eHealth technology. Further research is needed to determine the independent effects of these technologies on weight management for studies employing multimodal intervention methods.

#### Effective Components of eHealth Interventions

The growth in eHealth apps is related to the underlying presumption that their use will be associated with lower health care costs and improvements in health outcomes, particularly when focusing on the prevention of diseases and promotion of healthy lifestyles. Although eHealth technologies have the potential to improve prenatal health care delivery by providing frequent, interactive, and personalized information to broad populations in *real time*, there is a risk that the app may not be effective or could potentially result in harm to the mother and her unborn child. Thus, it is critical that eHealth intervention technologies be designed using an evidence-based approach and tested/evaluated with the addition of appropriate safeguards to ensure safety of the participants before being implemented into widespread use among the general population. This may include the performance of clinical trials that use a data safety and monitoring committee who will intervene in the occurrence of increased adverse events within a study.

Participant engagement is also critical to the success of any eHealth intervention. To date, technology-based weight management approaches have been well accepted in nonpregnant populations [7] and postpartum populations with up to 74% of postpartum women accessing and reviewing weight management materials immediately after receiving the resources in one study [12]. Studies have reported significant variability in the number of intervention participants that read and respond to study-based text messages, similar to the postpartum participants receiving eHealth interventions. Although the findings from this review suggest that multicomponent interventions (ie, combined focus on both nutrition and physical activity) resulted in more favorable weight management during pregnancy and postpartum, it is difficult to ascertain which component attributed to the observed effect or whether it is related to the entire “bundle” of interventions. Moreover, not all eHealth components are considered as useful or desired by participants within a weight management intervention. For example, only 14% of postpartum women utilized an online forum for interacting with other participants for peer support [34]. Consequently, before implementing an intervention of this type, investigators must carefully consider the design and features of the eHealth intervention for their target population. This includes ensuring the use of both effective and appropriate strategies and frameworks to provide reasonable engagement and adherence both in the short-term with long-term follow-up to determine whether these behaviors that are the targets of such interventions have lasting effects.

Recognizing the importance and value of patient engagement, the Canadian Institutes of Health Research (CIHR) examined a strategy for patient-oriented research (SPOR) where patients, researchers, health care providers, and decision makers work together to build a sustainable, accessible, and equitable health care system [43]. Applying this principle of patient engagement to the development of new eHealth apps is beneficial and necessary [44,45]. Including pregnant and postpartum women, whether during the initial app developmental process or through preliminary focus group trials, would provide tangible feedback during this critical period in areas such as GWG, physical activity, sleep, and nutrition. Ensuring that embedded tools and features are clear and easily accessible for various levels of literacy and digital experience is also a requirement [46]. For eHealth apps to gain traction and thus reach the widest audiences, endorsement from clinical stakeholders and health care providers will likely also be necessary [47].

### Strengths and Limitations

This review was conducted through the use of a comprehensive search designed to identify high-quality evidence on eHealth technologies on weight management in pregnant and postpartum women. As eHealth technologies are a novel yet growing area, only 10 studies of relatively small sample sizes were eligible for inclusion. Given the limited number of participants, the meta-analyzed results, while promising, must be interpreted with caution until further studies are conducted. Seven of these included studies were “unclear” risk of bias because of poorly reported methodologies, two were deemed to have high risk of bias, and only one was low risk of bias. In addition, the studies were conducted in the United States, Spain, and the United Kingdom, which may potentially limit the generalizability to the rest of the world. This study is timely as, at present, there are four registered clinical trials investigating the use of eHealth technologies in targeting weight management or lifestyle behaviors in pregnancy (Trial registration: ClinicalTrials.gov NCT02229708, NCT01948323, NCT01461707, NCT01610752), which will help to further inform this important area.

### Comparison With Existing Literature

O’Brien et al [30] previously conducted a technology-based systematic review in healthy pregnant women and reported that while these technologies have the potential to be helpful as a health care tool, further evidence in the form of RCTs is needed to determine the efficacy of mobile and other health technologies. However, this review included four ongoing studies (with no data) in addition to three published studies. Thus, a meta-analysis was not conducted as a result of the heterogeneity of their participant population. Lastly, a review of the quality of the evidence was not presented.

### Conclusions

Enhanced prenatal care has been identified as one of the most important strategies for preventing obesity and future chronic diseases [48]. As the importance of excessive GWG and postpartum weight retention on cardiometabolic risks in mothers and their offspring gain more clinical attention, this review suggests that weight management in women during pregnancy and the postpartum period may be enhanced through the use of eHealth technologies. The widespread availability and adaptability of eHealth technologies provides a novel widely available platform for delivering information and guidance on weight management during these critical periods. As intensive in-person interventions are impractical within most health care systems, innovative and scalable approaches for the management of weight during these important life periods are needed [29]. Although eHealth technologies demonstrate a promising and pragmatic approach to delivering health care advice and support for weight management, more comprehensive research with larger sample sizes, comprehensive outcome measures, and longer follow-up periods, is required to determine the optimal levels of eHealth intervention support, intensity, and duration during pregnancy and the postpartum period. Moreover, further investigation is needed to determine whether the effectiveness of eHealth interventions is modified through in-person contact with a health care professional. Overall, further research is necessary before widespread adoption of these eHealth interventions.

## Appendix

Multimedia Appendix 1 Pregnancy technology and weight loss search strategy.

Multimedia Appendix 2 Characteristics from the 10 selected studies summarizing the study objective, methods, participants, intervention, and risk of bias.

## Abbreviations

BMI: body mass index
CCT: clinical controlled trial
CDSR: Cochrane database of systematic reviews
CENTRAL: Cochrane Central Register of Controlled Trials
CIHR: Canadian Institutes of Health Research
CINAHL: Cumulative Index to Nursing and Allied Health Literature
DPP: Diabetes Prevention Program
eHealth: electronic health
GWG: gestational weight gain
HbA1C: glycated hemoglobin
IOM: Institute of Medicine
MeSH: MEDLINE database with medical subject headings
OR: odds ratio
PPAQ: pregnancy physical activity questionnaire
PRISMA: preferred reporting items for systematic reviews and meta-analysis
RCT: randomized controlled trial
SMS: short message service
SPOR: strategy for patient-oriented research
SD: standard deviation
UNDP: United Nations Millennium Development Goals
WHO: World Health Organization
