# Supplementary material for: The Effectiveness of eHealth Technologies on Weight Management in Pregnant and Postpartum Women: Systematic Review and Meta-Analysis
Source: J Med Internet Res. 2017 Oct 13;19(10):e337. doi: 10.2196/jmir.8006 (PMC5660296; doi:10.2196/jmir.8006)
Supplement: Multimedia Appendix 1 [file jmir_v19i10e337_app1.pdf]

Appendix 1. Pregnancy technology and weight loss search strategy.

Medline OVID (July 5 2016)

1. exp Pregnancy/
2. Postpartum Period/
3. perinatal care/ or postnatal care/ or "Prenatal Care"/
4. (pregnant or pregnancy).tw.
5. (postpartum or post partum or post natal or postnatal or perinatal).tw.
6. or/1-5
7. exp Exercise/
8. exp Diet/
9. body weight changes/ or weight loss/ or exp overweight/
10. (diet* or nutrition or "physical activity" or exerci*).ti,ab.
11. fitness.tw. or weight.ti.
12. exp Nutrition Therapy/
13. or/7-12
14. 6 and 13
15. Diabetes, Gestational/pc [Prevention & Control]
16. gestational diabetes.tw.
17. 15 or 16
18. 14 or 17
19. exp Cellular Phone/ or exp Internet/ or Computers/ or Computers, Handheld/ or Software/ or Computer-Assisted Instruction/ or Wireless Technology/ or ("cellular phone" or cell phone? or mobile or internet or web or computer-based or website or tablet or PDA or messaging or online).ti,ab.
20. electronic mail/ or text messaging/ or Telemedicine/
21. (blog or twitter or snapchat or social media or instragram or facebook or ehealth).tw.
22. (smartphone or smart phone or android or iphone or ipad).ti,ab.
23. "web 2.0".tw.
24. "web 3.0".tw.
25. or/19-24
26. 18 and 25
27. limit 26 to (clinical study or clinical trial, all or clinical trial, phase i or clinical trial, phase ii or clinical trial, phase iii or clinical trial, phase iv or clinical trial or comparative study or controlled clinical trial or meta analysis or pragmatic clinical trial or randomized controlled trial or systematic reviews)
28. (random* or systematic review or metaanalysis or meta-analysis).tw.
29. ((controlled or clinical or pilot) adj4 (study or trial)).tw.
30. (before adj2 after).tw.
31. ((control or comparison or reference) adj2 group*).ab.
32. 28 or 29 or 30 or 31

33. 26 and 32
34. 27 or 33
35. limit 34 to yr="1990 -Current"
36. remove duplicates from 35

Embase OVID (July 5 2016)

1. exp pregnancy/
2. postnatal care/ or puerperium/
3. prenatal care/
4. perinatal care/ or maternal care/
5. (pregnant or pregnancy).tw.
6. (postpartum or post partum or post natal or postnatal or perinatal).tw.
7. or/1-6
8. (diet* or nutrition or "physical activity" or exerci*).ti,ab.
9. exp exercise/
10. exp physical activity/
11. exp diet/
12. exp diet therapy/ or exp diet restriction/
13. nutrition/ or diet/ or diet therapy/ or dietary intake/ or food intake/ or maternal nutrition/ or nutrient management/ or nutrition education/ or nutritional assessment/ or nutritional counseling/ or nutritional health/
14. fitness.tw. or weight.ti.
15. body weight/ or weight change/ or weight control/ or weight fluctuation/ or weight gain/ or exp weight reduction/ or exp body weight management/
16. or/8-15
17. 7 and 16
18. pregnancy diabetes mellitus/
19. gestational diabetes.tw.
20. 18 or 19
21. 17 or 20
22. exp mobile application/ or telemedicine/ or exp computer program/ or exp microcomputer/ or exp mobile phone/ or exp Internet/ or exp personal digital assistant/
23. e-mail/ or social media/ or text messaging/ or videoconferencing/ or webcast/ or wireless communication/
24. ("cellular phone" or cell phone? or mobile or internet or web or computer-based or website or tablet or PDA or messaging or online).ti,ab.
25. (blog or twitter or snapchat or social media or instragram or facebook or ehealth).tw.
26. ("web 2.0" or "web 3.0").tw.
27. (smartphone or smart phone or android or iphone or ipad).ti,ab.
28. or/22-27
29. 21 and 28

30. limit 29 to (meta analysis or "systematic review")
31. exp Randomized Controlled Trials as Topic/
32. "Randomized Controlled Trial (topic)"/
33. Randomized Controlled Trial/
34. Randomization/
35. Random Allocation/
36. Double-Blind Method/
37. Double Blind Procedure/
38. Double-Blind Studies/
39. Single-Blind Method/
40. Single Blind Procedure/
41. Single-Blind Studies/
42. (random* or sham or placebo*).ti,ab,hw.
43. ((singl* or doubl*) adj (blind* or dumm* or mask*)).ti,ab,hw.
44. ((tripl* or trebl*) adj (blind* or dumm* or mask*)).ti,ab,hw.
45. (random* or systematic review or metaanalysis or meta-analysis).tw.
46. ((controlled or clinical or pilot) adj4 (study or trial)).tw.
47. (before adj2 after).tw.
48. ((control or comparison or reference) adj2 group*).ab.
49. or/31-48
50. 29 and 49
51. 30 or 50
52. limit 51 to yr="1990 -Current"
53. remove duplicates from 52

PsycINFO OVID (July 5 2016)

1. pregnancy/ or perinatal period/ or postnatal period/
2. prenatal care/
3. (pregnant or pregnancy).tw.
4. (postpartum or post partum or post natal or postnatal or perinatal).tw.
5. or/1-4
6. exp diets/
7. exp exercise/ or physical fitness/ or weight control/
8. body weight/ or weight gain/ or weight loss/
9. nutrition/ or diets/
10. (diet* or nutrition or "physical activity" or exerci*).ti,ab.
11. fitness.tw.
12. weight.ti.
13. or/6-12
14. 5 and 13
15. gestational diabetes/

16. gestational diabetes.tw.
17. 15 or 16
18. 14 or 17
19. ("cellular phone" or cell phone? or mobile or internet or web or computer-based or website or tablet or PDA or messaging or online).ti,ab.
20. exp Internet/ or exp Cellular Phones/ or exp Computer Applications/ or exp Mobile Devices/ or exp Telemedicine/
21. exp electronic communication/
22. social media/ or online social networks/ or blog/
23. (blog or twitter or snapchat or social media or instragram or facebook or ehealth).tw.
24. text messaging/
25. (smartphone or smart phone or android or iphone or ipad).ti,ab.
26. "web 2.0".tw.
27. "web 3.0".tw.
28. or/19-27
29. 18 and 28
30. limit 29 to ("0200 book" or "0240 authored book" or "0280 edited book" or "0400 dissertation abstract")
31. 29 not 30
32. (random* or systematic review or metaanalysis or meta-analysis).tw.
33. ((control or comparison or reference) adj2 group*).ab.
34. (before adj2 after).tw.
35. ((controlled or clinical or pilot) adj4 (study or trial)).tw.
36. or/32-35
37. 31 and 36
38. limit 37 to yr="1990 -Current"
39. remove duplicates from 38

Cochrane Central OVID (July 5, 2016)

1. exp Pregnancy/
2. Postpartum Period/
3. perinatal care/ or postnatal care/ or "Prenatal Care"/
4. (pregnant or pregnancy).tw.
5. (postpartum or post partum or post natal or postnatal or perinatal).tw.
6. or/1-5
7. exp Exercise/
8. exp Diet/
9. body weight changes/ or weight loss/ or exp overweight/
10. (diet* or nutrition or "physical activity" or exerci*).ti,ab.
11. fitness.tw. or weight.ti.
12. exp Nutrition Therapy/

13. or/7-12
14. 6 and 13
15. Diabetes, Gestational/pc [Prevention & Control]
16. gestational diabetes.tw.
17. 15 or 16
18. 14 or 17
19. exp Cellular Phone/ or exp Internet/ or Computers/ or Computers, Handheld/ or Software/ or Computer-Assisted Instruction/ or Wireless Technology/ or ("cellular phone" or cell phone? or mobile or internet or web or computer-based or website or tablet or PDA or messaging or online).ti,ab.
20. electronic mail/ or text messaging/ or Telemedicine/
21. (blog or twitter or snapchat or social media or instragram or facebook or ehealth).tw.
22. (smartphone or smart phone or android or iphone or ipad).ti,ab.
23. "web 2.0".tw.
24. "web 3.0".tw.
25. or/19-24
26. 18 and 25
27. limit 26 to yr="1990 -Current"
28. remove duplicates from 27

Cochrane Database of Systematic Reviews OVID (July 5 2016)

1. (pregnant or pregnancy).tw.
2. (postpartum or post partum or post natal or postnatal or perinatal).tw.
3. 1 or 2
4. (diet* or nutrition or "physical activity" or exerci*).ti,ab.
5. fitness.tw. or weight.ti.
6. lifestyle.tw.
7. 4 or 5 or 6
8. 3 and 7
9. gestational diabetes.tw.
10. 8 or 9
11. ("cellular phone" or cell phone? or mobile or internet or web or computer-based or website or tablet or PDA or messaging or online).ti,ab.
12. (blog or twitter or snapchat or social media or instragram or facebook or ehealth).tw.
13. (smartphone or smart phone or android or iphone or ipad).ti,ab.
14. "web 2.0".tw.
15. "web 3.0".tw.
16. 11 or 12 or 13 or 14 or 15
17. 10 and 16

CINAHL EBSCO (July 5 2016)

#	Query	Limiters/Expanders
S32	S25 AND S30	Limiters - Published Date: 19900101-20161231<br>Search modes - Boolean/Phrase
S31	S25 AND S30	Search modes - Boolean/Phrase
S30	S26 OR S27 OR S28 OR S29	Search modes - Boolean/Phrase
S29	TX ((control or comparison or reference) N2 group*)	Search modes - Boolean/Phrase
S28	TX (before N2 after)	Search modes - Boolean/Phrase
S27	TX ((controlled or clinical or pilot) N4 (study or trial))	Search modes - Boolean/Phrase
S26	(MH"Randomized Controlled Trials") or (MH"Clinical Trial") or (MH"Control Group") or (MH"Nonequivalent Control Group") or (MH"Systematic Review") or (MH"Meta-Analysis") or (MH"Matched-Pair Analysis") or (TI("controlled before-and-after" or "historically controlled" or "systematic review" or "meta-analysis")) or (AB("controlled before-and-after" or "historically controlled" or "systematic review" or "meta-analysis"))	Search modes - Boolean/Phrase
S25	S15 AND S24	Search modes - Boolean/Phrase
S24	S16 OR S17 OR S18 OR S19 OR S20 OR S21 OR S22 OR S23	Search modes - Boolean/Phrase
S23	TX ("web 2.0" or "web 3.0").	Search modes - Boolean/Phrase
S22	(MH "Electronic Bulletin Boards") OR (MH "Electronic Mail")	Search modes - Boolean/Phrase
S21	(MH "Wireless Communications")	Search modes - Boolean/Phrase
S20	(MH "Text Messaging") OR (MH "Instant Messaging")	Search modes - Boolean/Phrase
S19	TX (smartphone or smart phone or android or iphone or ipad).	Search modes - Boolean/Phrase

S18	TX (blog or twitter or snapchat or social media or instragram or facebook or ehealth)	Search modes - Boolean/Phrase
S17	(MH "Social Media")	Search modes - Boolean/Phrase
S16	(MH"Internet+") or (MH"World Wide Web") or (MH"Wireless Communications") or (MH"Text Messaging") or (MH"Instant Messaging") or (MH"Computers,Hand-held") or (MH"Software") or (MH"Computer Assisted Instruction") or (TI("cellular phone" or mobile or internet or computers or website or tablet or PDA or messaging)) or (AB("cellular phone" or mobile or internet or computers or website or tablet or PDA or messaging))	Search modes - Boolean/Phrase
S15	S11 OR S14	Search modes - Boolean/Phrase
S14	S12 OR S13	Search modes - Boolean/Phrase
S13	(MH "Diabetes Mellitus, Gestational/PC/DH") OR (MH "Pregnancy in Diabetes/DH/PC")	Search modes - Boolean/Phrase
S12	"gestational diabetes"	Search modes - Boolean/Phrase
S11	S5 AND S10	Search modes - Boolean/Phrase
S10	S6 OR S7 OR S8 OR S9	Search modes - Boolean/Phrase
S9	TI weight OR TX fitness	Search modes - Boolean/Phrase
S8	(MH"Body Weight") or (MH"Weight Loss") or (MH"Weight Gain") or (MH"Weight Reduction Programs") or (MH"Weight Control") or TI((weight or "weight maintenance" or "weight reduction" or "weight loss" or "body weight" or "weight change" or "weight control" or "weight gain" or "weight management")) or AB((weight or "weight maintenance" or "weight reduction" or "weight loss" or "body weight" or "weight change" or "weight control" or "weight gain" or "weight management"))	Search modes - Boolean/Phrase
S7	(MH "Nutrition Services") OR (MH "Nutritional Counseling")	Search modes - Boolean/Phrase
S6	(MH"Diet") or (MH"Physical Activity") or (TI(diet or nutrition or "physical activity" or exercise)) or (AB(diet or nutrition or "physical activity" or exercise))	Search modes - Boolean/Phrase
S5	S1 OR S2 OR S3 OR S4	Search modes -

		Boolean/Phrase
S4	TX (postpartum or post partum or post natal or postnatal or perinatal)	Search modes - Boolean/Phrase
S3	TX (pregnant or pregnancy)	Search modes - Boolean/Phrase
S2	(MH "Postnatal Care") OR (MH "Prenatal Care") OR (MH "Perinatal Care")	Search modes - Boolean/Phrase
S1	(MH "Pregnancy")	Search modes - Boolean/Phrase
