# Supplementary material for: The Effectiveness of eHealth Technologies on Weight Management in Pregnant and Postpartum Women: Systematic Review and Meta-Analysis
Source: J Med Internet Res. 2017 Oct 13;19(10):e337. doi: 10.2196/jmir.8006 (PMC5660296; doi:10.2196/jmir.8006)
Supplement: Multimedia Appendix 2 [file jmir_v19i10e337_app2.pdf]

Appendix 2. Characteristics from the 10 selected studies summarizing the study objective, methods, participants, intervention, and risk of bias.

**Study, Location**	Carral (2015), Spain [40]
**Objective**	The aim of this study was to examine the impact of a web-based telemedicine system for monitoring glucose control in pregnant women with diabetes on healthcare visits, metabolic control, and pregnancy outcomes.
**Methods**	Design: CCT
	Selection: 104 pregnant women with diabetes (77 with gestational diabetes mellitus (GDM), 16 with type 1 diabetes mellitus, and 11 with type 2 diabetes mellitus) were consecutively recruited among patients assisted at the Gestational Diabetes Unit (GDU) of the Endocrinology Department of Puerto Real University Hospital (Cadiz, Spain).
	Inclusion criteria: Over 18 years with diabetes mellitus diagnosed before pregnancy (PGDM) or diabetes diagnosed in the current pregnancy (GDM) referred to the GDU before week 30 of pregnancy.
**Participants**	Sample: N = 104
	Intervention: n = 40; Control: n = 64
	Follow-up: Intervention: n = 36; Control: n = 53
	Mean Age (SD): Intervention: 34.9 ± 3.9y; Control: 33.2 ± 4.9y
**Intervention**	Intervention duration: 18 months
	Type of intervention: Web-based with a focus on diet and nutrition.
	Description of intervention: The telemedicine group (TMG), was monitored by both more spaced GDU visits and a web-based telemedicine system.
	Description of control: The control group (CG), which was managed only by regular visits to hospital (to the GDU and a nurse educator).
	Length of follow-up: 6-12 weeks postpartum
	Outcome: Maternal - time of delivery (gestational weeks), delivery before 37 weeks, caesarean delivery, weight gain, GDM with insulin treatment, pregnancy-induced hypertension (HT), hospital stay (days); Neonatal - miscarriages, birth weight, large for gestational age, small for gestational age, newborn males, hypoglycemia, other neonatal complications; Health visits to GDU - nurse educator, obstetric service, hospital emergency, general practitioner, ambulatory nurse, online.
**Risk of Bias**	Sequence Generation: High

	Allocation Concealment: High
	Blinding of Patient/Participants and Providers/Personnel: High
	Selective Reporting: Low
	Other Bias: Low
	Overall Risk: High

**Study, Location**	Colleran (2012), USA [32]
**Objective**	Use the MyPyramid Menu Planner and individualized diet counseling to improve diet quality and pattern of food consumption in overweight/obese postpartum women.
**Methods**	Design: RCT
	Selection: Targeted recruitment at prenatal classes and out-patient obstetrician offices.
	Inclusion/Exclusion criteria: *Inclusion* - 23-37y, delivered full-term infant (>37w), self-report postpartum BMI of 25-30 kg/m^2, breastfeeding, less than 3w postpartum, <3 days/w of structured physical activity, physician clearance. *Exclusion* - smoking, c-section, hormone-affected medical conditions, contraindications to exercise.
**Participants**	Sample: N = 31
	Intervention: n = 16; Control: n = 15
	Follow-up: Intervention: n = 14; Control: n = 13
	Mean Age (SD): Intervention: 31.9 ± 3.1y; Control: 30.3 ± 0.8y
**Intervention**	Intervention duration: 16 weeks, beginning from 4-20 weeks postpartum.
	Type of intervention: Intensive lifestyle intervention targeting weight loss.
	Description of intervention:
	Exercise – strength training 3 times per week, walking 10,000 steps or 3,000 aerobic steps 5 times per week.
	Diet – reduce dietary intake by 500 kcal/day below energy requirements (dietary reference intakes). Received a multivitamin supplement.
	Research assistants traveled to the participant’s home up to 3 times per week to deliver the intervention (exercise) and for face-to-face consultation. At least one face-to-face contact was held each week.

	Description of control: Control participants were asked not to participate in a structured exercise program during the 16 week study. Received 2 educational handouts at 9 and 16 weeks postpartum. Received a multivitamin supplement. Control group was not intensity-matched.
	Length of follow-up: 16 weeks
	Outcome: Significant difference (7%) in weight loss between the intervention group (5.8 ± 3.5 kg) and the control group (1.6 ± 5.4 kg). The number of weeks that MyPyramid Menu Planner was utilized by participants was not associated with weight loss.
**Risk of Bias**	Sequence Generation: Unclear
	Allocation Concealment: Unclear
	Blinding of Patient/Participants and Providers/Personnel: Unclear
	Selective Reporting: Low
	Other Bias: Unclear (industry sponsored/funded but no explicit details about involvement)
	Overall Risk: Unclear

**Study, Location**	Herring (2014), USA [38]
**Objective**	To examine the feasibility, acceptability, and initial efficacy of a technology-based weight loss intervention for urban, low-income mothers.
**Methods**	Design: RCT
	Selection: Women were recruited from the waiting rooms of 2 large outpatient practices (obstetrics and pediatrics), which served primarily Medicaid-insured patients in Philadelphia, PA.
	Inclusion/Exclusion criteria: *Inclusion* - eligibility criteria included: 1) age ≥ 18 years; 2) singleton infant delivered within the last 2 weeks to 12 months; 3) early pregnancy (first trimester) BMI ≥ 25 kg/m^2 via prenatal records; 4) weight at enrollment that exceeded early pregnancy weight by at least 5 kg; 5) cell phone ownership with unlimited text messaging; and 6) member of Facebook. *Exclusion* - included current tobacco use and any history of cardiac, gastrointestinal, cognitive, or psychiatric disorders.
**Participants**	Sample: N = 18
	Intervention: n = 9; Control: n = 9
	Mean Age (SD): Intervention: 24.2 ± 5.1y; Control: 24.2 ± 5.1y

	Follow-up: Intervention: n = 8; Control: n = 9
**Intervention**	Intervention duration: 14 weeks
	Type of intervention: Web-based and text-messaging focusing on nutrition and physical activity.
	Description of intervention: Using the interactive obesity treatment approach as our guide, the intervention (Healthy4Baby) was designed to create an energy deficit sufficient to produce weight loss by focusing on the modification of evidence-based, weight-related lifestyle behaviors.<br><br>A set of 6 empirically supported weight-related behavior change strategies were identified and prioritized that were relevant to the patient population, could be communicated simply, and were easily self-monitored through text messaging.
	Description of control: Participants randomized to usual care received the current standard of care offered to postpartum mothers from their primary care providers or through the Special Supplemental Nutrition Program for women, infants, and children (WIC). Often, usual care meant one visit over the entire first postpartum year with their physicians (typically at 6 to 8 weeks postpartum), at which time providers screened for depression and counselled new mothers about breastfeeding and birth control. All mothers in the study received nutrition counseling and food/beverage vouchers from WIC, although WIC visit frequency varied among participants from monthly to every 3 months over the first year postpartum.
	Length of follow-up: 14 weeks
	Outcome: Change in body weight.
**Risk of Bias**	Sequence Generation: Low
	Allocation Concealment: Low
	Blinding of Patient/Participants and Providers/Personnel: High
	Selective Reporting: Low
	Other Bias: Low
	Overall Risk: Unclear

**Study, Location**	Herring (2016), USA [36]
**Objective**	This study evaluated whether a technology-based behavioral intervention could decrease the proportion of African American women with overweight

	or obesity who exceeded Institute of Medicine (IOM) guidelines for gestational weight gain.
**Methods**	Design: RCT
	Selection: Participants were 66 pregnant women who were either overweight or obese were recruited from two large outpatient obstetric practices at Temple University between 2013 and 2014. Study staff used Temple's electronic medical record to identify potential participants by BMI and age, and then approached them in waiting rooms to evaluate trial interest.
	Inclusion/Exclusion criteria: *Inclusion* - age 18 years; self-identification as African American; gestational age <20 weeks; first trimester BMI 25-45 kg/m^2; Medicaid recipient (income proxy); cell phone ownership with unlimited text messaging; and Facebook member. *Exclusion* - women were excluded if they had conditions requiring specialized nutritional care (e.g., bariatric surgery), endorsed current tobacco use, or multifetal gestation.
**Participants**	Sample: N = 66
	Intervention: n = 33; Control: n = 33
	Follow-up: Intervention: n = 27; Control: n = 29
	Mean Age (SD): Intervention: 25.9 ± 4.9y; Control: 25.0 ± 5.7y
**Intervention**	Intervention duration: 13 weeks
	Type of intervention: Text messaging with a focus on nutrition and physical activity.
	Description of intervention: Intervention components. Skills training and support were delivered through three mechanisms: 1) Skills training and self-monitoring texts with personalized feedback. Participants received daily text messages tailored to each behavioral goal to build skills and self-efficacy (e.g., One mom says, 'Snacking at night will just give me heartburn. But if it's yogurt, then I'll be good. I don't eat any junk late at night'). Participants also received self-monitoring texts three to four times weekly to probe about behavioral adherence. Text message prompts in the morning (e.g., "Please text us total # junk and grease u had yesterday.") were followed by immediate personalized automatic feedback to reinforce successes and/or offer support (e.g., "U had 0 junk and grease foods. Ur really working toward growing a healthy baby! Keep eating fruits and veggies, they're the healthiest!"). As an incentive, participants received raffle entries for responding to self-monitoring text prompts; an automated computer program randomly chose monthly $25 gift card winners. 2) Facebook. Participants were enrolled in a private Facebook group to provide a forum for support and additional behavioral skills training via links to websites and videos. Participants were encouraged to "like" weekly coach posts and provide updates. To ensure confidentiality, this group was by invitation only and had

	a generic name (e.g., “Pregnant Moms Temple”); information about privacy settings was also supplied. 3) Health coach calls. A bachelor’s level health coach, trained in methods of behavioral weight control, delivered 15 to 20 min counselling calls to participants weekly for the first two study weeks and then twice monthly thereafter. Counselling calls were designed to reinforce skills and provide the opportunity to problem-solve through barriers. To increase intervention fidelity, calls were scripted and audiotaped; 10% were reviewed at research meetings, with discussion and role-play retraining as needed. A web application was used for note taking and storage of process data (e.g., date/time of call, call attempts, disposition).
	Description of control: Women randomized to usual care received standard obstetrical care at Temple University, which included: 1) an initial visit in the first-trimester, during which obstetric providers completed a comprehensive patient history, physical exam, ultrasound, and blood work; 2) follow-up visits monthly until week 24 and every 2 to 3 weeks until week 36, where providers assessed patient weight, blood pressure, urine protein, and fetal heart rate; and 3) weekly visits from week 36 until delivery. Information from the American College of Obstetricians and Gynecologists (ACOG) about optimal weight gain during pregnancy was also supplied.
	Length of follow-up: from study entry to 36 weeks gestation.
	Outcome: The primary outcome measure for this study was the proportion of women with excessive gestational weight gain based on the 2009 IOM guidelines for full term, singleton pregnancies, neonatal weight outcomes and maternal obstetric outcomes.
**Risk of Bias**	Sequence Generation: Low
	Allocation Concealment: Low
	Blinding of Patient/Participants and Providers/Personnel: Unclear
	Selective Reporting: Low
	Other Bias: Low
	Overall Risk: Low

**Study, Location**	Kim (2012), USA [33]
**Objective**	To determine the feasibility of a web-based intervention targeting self-efficacy behaviors for increasing physical activity (steps per day). The primary outcome variable was change in glucose concentration between the control and intervention group.
**Methods**	Pilot study. Low-intensity pedometer based intervention.

	Design: RCT
	Selection: Women with previous medical history (Hx) of GDM were identified in medical records (targeted mailing) or physician offices (patient referrals).
	Inclusion/exclusion criteria: *Inclusion*- Women with previous Hx of GDM within past 3y, >18y, <150 minutes per week of exercise, not currently pregnant, non-diabetic (confirmed by 75g OGTT), fluent in English, email address and access to Windows XP/Vista, medical clearance from medical provider. *Exclusion* - use of metformin or oral glucocorticoids.
**Participants**	Sample: N = 49
	Intervention: n = 21; Control: n = 28
	Follow-up: Intervention: n = 19; Control: n = 23
	Mean Age (SD): Intervention: 35.9 ± 3.3y; Control: 35.5 ± 4.7y
**Intervention**	Intervention Duration: 13 weeks
	Type of intervention: Low-intensity web-based intervention to increase physical activity.
	Description of intervention: Structured web-based intervention using pedometers. Women received a pedometer and access to online curriculum. Pedometer data was downloaded and step data displayed (graphically and via text).
	Description of control: Received no educational materials during the intervention but received pedometer following completion of the study.
	Length of follow-up: Follow-up was completed at the end of the intervention (~13 weeks after baseline testing).
	Outcome: No significant differences between groups in behaviors, physical activity parameters, glucose, insulin, and weight.
**Risk of Bias**	Sequence Generation: Unclear
	Allocation Concealment: Low
	Blinding of Patient/Participants and Providers/Personnel: Low
	Selective Reporting: Low
	Other Bias: Low
	Overall Risk: Unclear

**Study,**	Nicklas (2014), USA [37]

**Location**	
**Objective**	To test the feasibility and effectiveness of a web-based lifestyle intervention based on the diabetes prevention program modified for women with recent gestational diabetes mellitus to reduce postpartum weight retention.
**Methods**	Design: RCT
	Selection: Postpartum women from the Diabetes in Pregnancy Program at Brigham and Women's Hospital.
	Inclusion /Exclusion criteria: *Inclusion:* women aged 18–45 years, with GDM in their most recent pregnancy, body mass index (BMI) ≤24 kg/m^2; ≤22 kg/m^2 for Asian participants. *Exclusion:* women with BMI higher than 50 kg/m^2 because they would require a more intensive program, women delivering before 32 weeks of gestation and with net weight loss during pregnancy, women with a personal history of type 2 diabetes or bariatric surgery, women taking medications known to affect body weight, and women unable to read eighth grade-level English, or planning to move out of the area.
**Participants**	Sample: N = 75
	Intervention: n = 36; Control: n = 39
	Follow-up: Intervention = 33; Control = 35 (*the model imputed data for all participants)
	Mean Age (SD): Intervention: 33.6 ± 4.8y; Control: 33.3 ± 5.8y
**Intervention**	Intervention duration: 12 month
	Type of intervention: Web-based lifestyle modification with a focus on nutrition and physical therapy.
	Description of intervention: Participants allocated to the intervention were offered a web-based lifestyle modification program (Balance after Baby). We adapted the 16 core Diabetes Prevention Program modules to 12 core modules tailored for postpartum women with recent GDM. Adaptation included content review by a multidisciplinary team and by subject matter experts at the Massachusetts Department of Public Health, the Special Supplemental Nutrition Program for Women, Infants and Children, and the Centers for Disease Control and Prevention. Women in the Balance after Baby program were given a goal to return to pre-pregnancy weight over the study period. If participants were still overweight once they had reached their pre-pregnancy weight, they were encouraged to continue to lose weight with a goal of total 7% weight loss from the 6-week postpartum weight. The intervention program emphasized dietary choices that would transition readily from the pregnancy GDM diet, including lower glycemic index, higher fiber, and controlled portion sizes. The program recommended

	gradually increasing physical activity to 150 minutes per week or more, including resistance training. They suggested participants track diet and physical activity in log books and participate in telephone or e-mail sessions with their lifestyle coach, a licensed registered dietitian trained in patient-centered counseling. They encouraged women who were breastfeeding to continue. They created the secure password-protected Balance after Baby web site, including animated videos narrated by a study physician. They asked participants to watch one module each week for the first 12 weeks, and there were six optional “Balance after Core” modules available. The web site provided secure communication with the lifestyle coach, forms to enter goals, weekly weight and physical activity, shopping lists, recipes, menu planning tips, exchange lists, and physical activity education. The breastfeeding section contained four additional breastfeeding modules and a mechanism to contact a lactation consultant.
	Description of control: Patients allocated to the control arm did not receive any additional information to support weight loss beyond the handout they received at recruitment.
	Length of follow-up: 6 and 12 month
	Outcome: The two primary outcomes were change in measured body weight at 12 months from 1) first postpartum measured weight and 2) self-reported pre-pregnancy weight. Compared baseline characteristics with Pearson’s Chi-square or Fisher’s exact tests for categorical variables, and t tests or Wilcoxon rank sum for continuous variables. Patients were categorized based on treatment allocation in an intent-to-treat fashion for analyses. Compared differences between groups over time for weight, BMI, fasting glucose, 2-hour glucose, and HbA1c.
**Risk of Bias**	Sequence Generation: Unclear
	Allocation Concealment: Low
	Blinding of Patient/Participants and Providers/Personnel: Low
	Selective Reporting: Low
	Other Bias: Unclear
	Overall Risk: Unclear

**Study, Location**	Pérez-Ferre (2010), Spain [39]
**Objective**	To evaluate the feasibility of a telemedicine system based on Internet and a short message service in pregnancy and its influence on delivery and neonatal outcomes of women with gestational diabetes mellitus (GDM).

**Methods**	Design: RCT
	Selection: Eligible women diagnosed as having GDM (Carpenter-Coustan criteria) before 28 weeks of gestation and referred to the Unit of Gestational Diabetes of the Hospital Cl´ınico Universitario San Carlos (HCSC) of Madrid, Spain, from June to December 2007 were invited to participate in the study. Sixteen women were excluded, 10 with inability to understand or to comply with the protocol and 6 women refused to participate. A total of 100 women gave their written informed consent and were allocated either to the intervention group (A), provided with a telemedicine system detailed below, or allocated to the control group (B) that was treated in accordance with our standard face-to-face monitoring outpatient protocol.
	Inclusion/Exclusion criteria: *Inclusion* - women diagnosed as having GDM (Carpenter-Coustan criteria) before 28 weeks of gestation. *Exclusion* - women were excluded if they were unable to understand or comply with the protocol.
**Participants**	Sample: N = 100
	Intervention: n = 50; Control: n = 50
	Follow-up: Intervention: n = 49; Control: n = 48
	Mean Age (SD): Intervention: 33.3 ± 5.58y; Control: 34.2 ± 5.18y
**Intervention**	Intervention duration: Between week 24-28 of pregnancy until delivery
	Type of intervention: Web-based and smartphone application with a focus on diet and nutrition.
	Description of intervention: The telemedicine system consists of a central database and peripheral units, with cellular phones and a glucometer capable of transmitting data via infrared port.<br><br>Each woman in the intervention group received a glucometer (Accu-Chek Compact Plus) with a cellular phone (Nokia E50-1), the latter with a preinstalled application that allows the transmission of capillary glucose values to the central database via short message service (SMS). This application has also an interface that allows the infrared transmission of the glucose values stored in the glucometer to the cellular phone. The system enables the patient to regularly transmit blood glucose values and also to maintain contact through short text messages with health professionals as required.
	Description of control: Patients in the control group were followed according to protocol for gestational diabetes at HCSC, including the same capillary blood glucose targets, and were given the opportunity to attend the outpatient clinic without prior appointment (non-scheduled visit) and bring in their logbook when their blood glucose values were above the objectives or for any queries regarding nutritional recommendations or insulin dose. The total

	number of patients' non-scheduled visits to the medical centre, loss of workdays, and the number of hospital admissions were regularly recorded.
	Length of follow-up: before 28 weeks of gestation (visit 1), and between 32–34 (visit 2), 36–38 (visit 3), and 39-40 weeks (visit 4). Body weight, blood pressure, HbA1c, and first morning urine sample albuminto-creatinine ratio were determined in each visit.
	Outcome: percentage of women achieving HbA1c values; pregnancy, delivery, and newborn data and outcomes.
**Risk of Bias**	Sequence Generation: Unclear
	Allocation Concealment: Unclear
	Blinding of Patient/Participants and Providers/Personnel: Unclear
	Selective Reporting: Low
	Other Bias: Low
	Overall Risk: Unclear

**Study, Location**	Pollak (2014), USA [34]
**Objective**	To test the feasibility, acceptability and efficacy of an individualized SMS texting intervention (PregCHAT) for managing gestational weight gain for overweight and obese pregnant women in comparison to a generalized texting intervention (Txt4baby).
**Methods**	Design: RCT
	Selection: Study staff reviewed medical records in prenatal clinics to determine potential eligible participants who were then approached by clinic staff to determine if they were interested in participating.
	Inclusion/exclusion criteria: *Inclusion:* age of ≥18y; English-speaking; registered for prenatal care at participating clinics; pre-pregnancy BMI of 25-40 kg/m^2; 12-21 weeks pregnant; having a cell phone with an unlimited texting plan for the next five months. *Exclusion:* pre-existing diabetes; limited mobility or inability to walk; impaired cognition or mental health with inability to provide consent.
**Participants**	Sample: N = 35
	Intervention: n = 23; Control: n = 12
	Follow-up: Intervention: n = 14; Control n = 9
	Mean Age overall (SD): Intervention: 29 ± 5y; Control: 32 ± 2y.

**Intervention**	Intervention Duration: Approximately 16 weeks
	Type of intervention: Low-intensity text-messaging intervention targeting nutrition and physical activity health behaviour goals.
	Description of intervention: A texting-based intervention that target four health behaviours goals (increase daily walking to 10,000 steps; avoid sweetened drinks; eat at least five fruits and vegetables each day; eliminating fast food intake); participants receive texts 3 times per week requesting information on meeting their goals, weight and feedback/progress on their goals; also receive a monthly message with information surrounding their gestational weight gain target.
	Description of control: Participants were assigned to the "Text4Baby" national program which provides three text messages per week on general pregnancy information.
	Length of follow-up: Follow-up was completed at 22 wks, 32 wks and at the end of pregnancy.
	Outcome: There was a non-significant difference of 2 lbs for the final follow-up weight at the end of pregnancy between the control and intervention groups.
**Risk of Bias**	Sequence Generation: Unclear
	Allocation Concealment: Unclear
	Blinding of Patient/Participants and Providers/Personnel: Unclear
	Selective Reporting: Low
	Other Bias: Low
	Overall Risk: Unclear

**Study, Location**	Smith (2016), USA [35]
**Objective**	This study's objective was to determine if a web-based behavioral intervention (BI) can prevent excessive gestational weight gain (GWG) by increasing physical activity (PA).
**Methods**	Design: RCT
	Selection: Participants were recruited by local prenatal clinics and a large partnering hospital. Additional recruitment strategies included e-mail list services, online advertisements, and fliers posted within the community.
	Inclusion/Exclusion criteria: *Inclusion* - only women with a history of participating in fewer than 3 sessions of exercise for 30 minutes or more per

	week, 16 for at least 6 months before conception were enrolled. Additional inclusion criteria included being 18 to 45 years old, speaking English, having regular Internet access, and being willing to walk 30 minutes on most days of the week if asked to do so. *Exclusion* - having a history of gestational diabetes mellitus, preeclampsia, or chronic disease (e.g., type 1 diabetes mellitus, heart disease, renal disease); being underweight; smoking during pregnancy; and having a condition or using a medication known to influence overall metabolism.
**Participants**	Sample: N = 51
	Intervention: n = 26; Control: n = 25
	Follow-up: Intervention: n = 24; Control: n = 21
	Mean Age (SD): Intervention: 29.7 ± 4.1y; Control: 29.6 ± 4.5y
**Intervention**	Intervention duration: 26 weeks
	Type of intervention: Web-based with a focus on exercise and physical activity.
	Description of intervention: BI participants had access to all of the website features, including the same diet and PA recommendations as UC, as well as exercise goal-setting modules, problem-solving modules, a journal, a calendar to track all exercise until delivery, and a community forum to interact with other participants in the BI (social support).
	Description of control: Participants receiving UC could only view general prenatal diet and PA recommendations including American College of Obstetricians and Gynecologists guidelines and benefits of PA during pregnancy.
	Length of follow-up: 14 and 24 weeks
	Outcome: Gestational Weight Gain Physical Activity Dietary Intake Assessment.
**Risk of Bias**	Sequence Generation: Low
	Allocation Concealment: Unclear
	Blinding of Patient/Participants and Providers/Personnel: High
	Selective Reporting: Low
	Other Bias: Low
	Overall Risk: Unclear

**Study,**	Soltani (2015), UK [41]

**Location**	
**Objective**	The maternal obesity management using mobile technology (MOMTech) study aimed at evaluating the feasibility of text messaging based complex intervention designed to support obese women (BMI ≥ 30) with healthier lifestyles and limit GWG.
**Methods**	Design: CCT
	Selection: Woman accessing the maternity units in Doncaster Royal Infirmary Hospital were invited to participate.
	Inclusion/Exclusion criteria: *Inclusion* - pregnant women with a BMI ≥30; aged 18 years; able to read and understand English language. *Exclusion* - history of complications such as diabetes, hypertension, antepartum haemorrhage, unexplained fetal loss/stillbirth, psychiatric illness, or a multiple pregnancy.
**Participants**	Sample: N = 28
	Intervention: n = 16; Control: n = 14
	Follow-up: Intervention; n = 14 ; Control: n = 14 (convenience sample for women who declined or dropped out of intervention
	Mean Age (SD): Intervention: 29.1 ± 5.4y; Control: 31.7 ± 5.8y
**Intervention**	Intervention duration: 26 weeks
	Type of intervention: Text messaging with a focus on weight management, physical activity and nutrition quality.
	Description of intervention: Participants received two daily text messages, supported by four appointments with healthy lifestyle midwife, diet and activity goal setting, and self-monitoring diaries. The comparison group were obese mothers who declined to participate but consented for their routinely collected data to be used for comparison. Postnatal interviews and focus groups with participants and the comparison group explored the intervention's acceptability and suggested improvements.
	Description of control: For data analysis purposes the 14 participating women in the intervention group were compared with those originally declined (6), those who changed their mind before written consent (8), and one who did not attend after consenting (1), making a comparison group (CG) of 15 in total.
	Length of follow-up: Immediate post intervention (at birth)
	Outcome: Gestational Weight Gain; Birthweight and Gestational age
**Risk of Bias**	Sequence Generation: High

	Allocation Concealment: High
	Blinding of Patient/Participants and Providers/Personnel: High
	Selective Reporting: Low
	Other Bias: High
	Overall Risk: High

BMI = body mass index
CCT = clinical controlled trial
GDM = gestational diabetes
GWG = gestational weight gain
Hx = history
MVPA = moderate-to-vigorous physical activity
PA = physical activity
RCT = randomized controlled trial
SD = Standard deviation
W = weeks
Y = years
75 g OGTT = 75 gram oral glucose tolerance test
